# The risk of preterm birth in vanishing twin: A multicenter prospective cohort study

**DOI:** 10.1371/journal.pone.0233097

**Published:** 2020-05-29

**Authors:** Ji Su Seong, You Jung Han, Min Hyoung Kim, Jae-Yoon Shim, Mi-Young Lee, Soo-young Oh, Joon Ho Lee, Soo Hyun Kim, Dong Hyun Cha, Geum Joon Cho, Han-Sung Kwon, Byoung Jae Kim, Mi Hye Park, Hee Young Cho, Hyun Sun Ko, Chan-Wook Park, Joong Shin Park, Jong Kwan Jun, Hyun Mee Ryu, Seung Mi Lee

**Affiliations:** 1 Department of Obstetrics and Gynecology, Seoul National University College of Medicine, Seoul, Korea; 2 Department of Obstetrics and Gynecology, CHA Gangnam Medical Center, CHA University, Seoul, Korea; 3 Department of Obstetrics and Gynecology, MizMedi Hospital, Seoul, Korea; 4 Department of Obstetrics and Gynecology, University of Ulsan College of Medicine, Asan Medical Center, Seoul, Korea; 5 Mirae & Heemang Obstetrics and Gynecology Clinic, Seoul, Korea; 6 Department of Obstetrics and Gynecology, Samsung Medical Center, Sungkyunkwan University School of Medicine, Seoul, Korea; 7 Department of Obstetrics and Gynecology, Institute of Women's Life Medical Science, Yonsei University College of Medicine, Yonsei University Health System, Seoul, Korea; 8 Department of Obstetrics and Gynecology, Korea University College of Medicine, Seoul, Korea; 9 Department of Obstetrics and Gynecology, Konkuk University School of Medicine, Seoul, Korea; 10 Department of Obstetrics and Gynecology, Seoul Metropolitan Government Seoul National University Boramae Medical Center, Seoul, Korea; 11 Department of Obstetrics and Gynecology, Ewha Womans University, Seoul, Korea; 12 Department of Obstetrics and Gynecology, Bundang CHA Hospital, CHA University, Pocheon-si, Korea; 13 Department of Obstetrics and Gynecology, Catholic University of Korea College of Medicine, Seoul, Korea; University of Mississippi Medical Center, UNITED STATES

## Abstract

**Objective:**

To evaluate not only the risk of total preterm birth (PTB) but also spontaneous preterm birth (sPTB) and indicated preterm birth (iPTB) in vanishing twin (VT).

**Study design:**

This is a secondary analysis of a multicenter prospective cohort study. In 12 different healthcare institutions, women with singleton pregnancies were enrolled in early pregnancy and followed up till delivery.

**Results:**

A total of 4,746 women were included in the final analysis, and. the frequency of VT was 1.1% (54/4746). VT group had a higher risk for total PTB (PTB<34 weeks, 2.1% vs. 14.8%, p<0.001; PTB<32 weeks, 1.6% vs. 13.0%, p<0.001; PTB<28 weeks, 0.9% vs. 13.0%, p<0.001) than singleton group. The VT group had increased risk for both sPTB and iPTB (<34 weeks, <32 weeks, and <28 weeks), and this increased risk for sPTB and iPTB in VT group remained significant even after controlling for confounders such as maternal age, parity, pre-pregnancy BMI, and mode of conception.

**Conclusion:**

Vanishing twin can be an independent risk factor for both sPTB and iPTB when compared with singleton pregnancy.

## Introduction

The “vanishing twin” is diagnosed when one twin “vanishes” or is lost in the first trimester. The prevalence of vanishing twin is reported up to 10 to 40 percent among twin pregnancies [1, 2]. It is already well known that its prevalence is higher in pregnant women who conceived after assisted reproductive technology (ART) [3] than in naturally pregnant women. However, the etiology of vanishing twin is still uncertain, and its exact prevalence is not well-known because ultrasonography at first trimester is not always possible in a clinical situation.

Traditionally, it was believed that this vanishing twin phenomenon do not affect the pregnancy outcomes of the remained co-twin [4]. However, a few recent studies described adverse obstetric outcomes such as preterm birth and low birth weight in vanishing twin [5–9], whereas other studies did not find differences between singleton pregnancy and vanishing twin [7, 10, 11]. In addition, there is a paucity of information regarding the risk of spontaneous preterm birth (sPTB) and indicated spontaneous preterm birth (iPTB) although it is plausible that vanishing twin might increase the risk of sPTB by an inflammatory condition [12].

The purpose of this prospective cohort study was to evaluate not only the risk of total PTB but also sPTB and iPTB in vanishing twin. The secondary outcome was to compare obstetric outcomes between singleton group and vanishing twin group.

## Materials and methods

### Study design

This is a secondary analysis of a multicenter prospective cohort study, which was designed to evaluate the cost-effectiveness of various prenatal test strategies for Down syndrome. From December 2016 to April 2018, in 12 different healthcare institutions, women with singleton pregnancies who are candidates for the identification of fetal aneuploidy were enrolled. The protocol of this prospective cohort study has been published [13]. In brief, all pregnant women who visited the participating hospitals before 24 weeks of gestations and counselled regarding fetal aneuploidy screening/diagnosis tests are invited to enroll in the current prospective study. Cases with spontaneous abortion before 14 weeks of gestation or pregnancy termination before fetal viability or cases that were lost to follow up were excluded. Patients were divided into two groups: vanishing twin (VT group) and singleton pregnancy originated from singleton gestation (singleton group). The risk of subsequent PTB including both sPTB and iPTB was compared between the two groups of cases. This study was approved by Institutional Review Board of Seoul National University Hospital. We follow the ethical standards for human experimentation established in the Declaration of Helsinki. Also, the patients provided their written consent for the collection and use of clinical information.

### Diagnosis of vanishing twin

At the time of enrollment, baseline pregnancy characteristics including the history of vanishing twin in index pregnancy were collected. Vanishing twin was diagnosed if two gestational sacs were noted in early ultrasonography, but one twin demise occurred before 14 weeks of gestation. Singleton pregnancy was defined as pregnancy originated from singleton gestational sac diagnosed in early ultrasonography. As the National Health Insurance Service of Korean government covers the cost of ultrasonography at least three times in the first trimester for all pregnant women, it is our routine practice to use ultrasonography in early pregnancy in all pregnant women.

### Obstetric outcomes

Spontaneous PTB (sPTB) was defined as PTB occurred after preterm labor, preterm premature rupture of membranes, or cervical insufficiency. Indicated PTB (iPTB) was defined as PTB because of maternal or fetal indication. The diagnosis of obstetric complications such as gestational hypertension, preeclampsia, gestational diabetes, preterm labor, preterm premature rupture of membranes, cervical insufficiency, and placental abruption was made by the attending physician. Small for gestational age (SGA) was defined as birth weight less than 10th percentile [14].

### Statistical analysis

The Statistics Package for Social Sciences (SPSS) was used for statistical analysis, and a P value of < .05 was considered statistically significant. Mann-Whitney *U*-test was used to compare differences of continuous parameters and Fisher’s exact test was used to compare proportions between the two groups. To determine vanishing twin is independently associated with the risk of preterm birth, multiple logistic regression analysis was used to adjust confounding variables, which were maternal age, parity, pre-pregnancy body mass index (BMI), and mode of conception. Confounding variables were chosen according to the result of univariate analysis as a risk factor for PTB (p<0.2).

## Results

**Fig 1:**
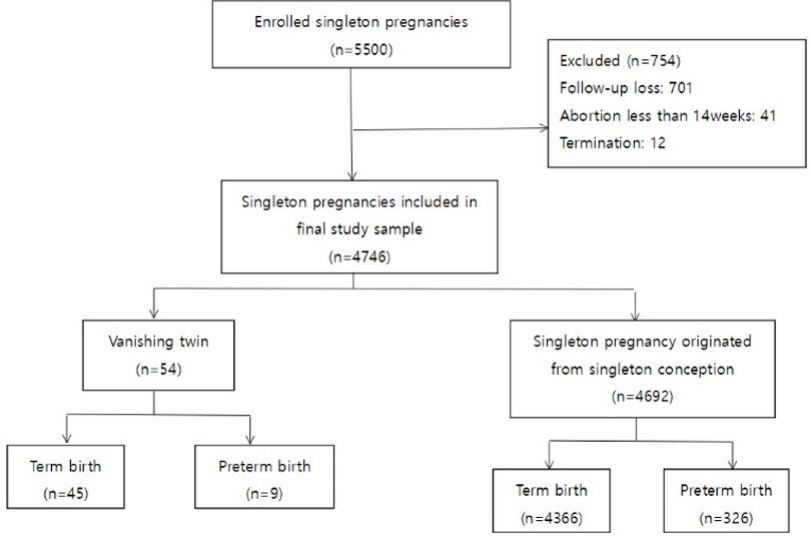
Study population.

During the study period, a total of 5,500 women were enrolled. After excluding cases with spontaneous abortion before 14 weeks of gestation (n = 41), cases with pregnancy termination before fetal viability (n = 12), and those cases who were lost to follow up (n = 701), the remained 4746 women were included in the final analysis (Fig 1).

Among the study population, the frequency of vanishing twin was 1.1% (54/4746). Maternal characteristics are summarized in Table 1. Women with vanishing twin were older and had higher pre-pregnancy BMI and higher frequency of nulliparity and conception after assisted reproduction.

**Table 1:** Characteristics of the study population.

	Singleton (n = 4,692)	Vanishing twin (n = 54)	p
Maternal age	33.00 (31–36)	36.00 (33–38)	<0.001
Pre-pregnancy BMI (n = 4573)	20.70 (19.20–22.60)	21.80 (20.40–23.20)	<0.05
Nulliparity	2831 (60.3%)	46 (85.2%)	<0.001
Conceived after assisted reproduction	528 (11.3%)	40 (74.1%)	<0.001

BMI, body mass index.

Data are presented as proportion (%) or median (IQR).

In the study population, the risks of preterm birth were 7.1% (335/4746) for PTB<37 weeks, 2.3% (107/4746) for PTB<34 weeks, 1.7% (80/4746) for PTB<32 weeks, and 1.1% (50/4746) for PTB<28 weeks (Table 2). The risks of PTB before 34 weeks, 32 weeks, and 28 weeks were higher in vanishing twin group than in singleton group, and this difference remained significant after confounding variables. In terms of etiology of PTB, the vanishing twin group had increased risk for both sPTB and iPTB (<34 weeks, <32 weeks, and <28 weeks), and this increased risk for sPTB and iPTB remained significant even after controlling for confounders including maternal age, parity, pre-pregnancy BMI, and mode of conception.

**Table 2:** Risk of preterm birth.

	Singleton (n = 4,692)	Vanishing twin (n = 54)	P	Adjusted OR* (95% CI)
**GA at delivery**	39.0 (38.3–39.9)	39.0 (38.0–39.9)	0.303	
**Birth weight (g) (n = 4675)**	3190 (2925–3450)	3220 (2893–3435)	0.925	
**Preterm birth**				
**<28 wks**	43 (0.9%)	7 (13.0%)	<0.001	8.02 (3.03–21.24)
Spontaneous PTB <28 wks	17 (0.4%)	3 (5.6%)	<0.005	8.53 (1.95–37.37)
Indicated PTB <28 wks	26 (0.6%)	4 (7.4%)	<0.001	7.14 (2.07–24.70)
**<32 wks**	73 (1.6%)	7 (13.0%)	<0.001	5.64 (2.24–14.19)
Spontaneous <32 wks	33 (0.7%)	3 (5.6%)	<0.01	4.89 (1.27–18.83)
Indicated PTB <32 wks	40 (0.9%)	4 (7.4%)	<0.005	5.73 (1.73–18.95)
**<34 wks**	99 (2.1%)	8 (14.8%)	<0.001	4.98 (2.12–11.71)
Spontaneous <34 wks	49 (1.0%)	4 (7.4%)	<0.005	4.88 (1.52–15.65)
Indicated PTB <34 wks	50 (1.1%)	4 (7.4%)	<0.005	4.47 (1.40–14.32)
**<37 wks**	326 (6.9%)	9 (16.7%)	<0.05	1.93 (0.90–4.14)
Spontaneous <37 wks	223 (4.8%)	5 (9.3%)	0.185	1.71 (0.65–4.50)
Indicated PTB <37 wks	103 (2.2%)	4 (7.4%)	<0.05	1.99 (0.66–6.00)

GA, gestational age; PTB, preterm birth.

* adjusted for maternal age, BMI, parity and assisted reproduction.

Table 3 shows the risk of obstetric complications in the study population. Cases with vanishing twin had a higher risk for fetal death in utero (FDIU), and this difference remained significant after adjustment for confounding variables. The risks of other obstetric complications such as preterm labor, pPROM, cervical insufficiency, gestational hypertension, preeclampsia, gestational diabetes, and placental abruption were higher in cases of vanishing twin group than those in singleton group, but this difference did not reach statistical significance. The risk of SGA was not different between the two groups of cases.

**Table 3:** Other obstetric outcomes.

	Singleton (n = 4,692)	Vanishing twin (n = 54)	P	Adjusted OR* (95% CI)
**Preterm labor**†	132/4655 (2.8%)	4/54 (7.4%)	0.069	2.34 (0.79–6.94)
**pPROM**†	368/4655 (7.9%)	6/54 (11.1%)	0.441	1.41 (0.58–3.41)
**Cervical insufficiency**†	7/4655 (0.2%)	1/54 (1.9%)	0.088	9.27 (0.76–112.47)
**Gestational hypertension**†	83/4655 (1.8%)	2/54 (3.7%)	0.255	0.90 (0.18–4.50)
**Preeclampsia**†	79/4655 (1.7%)	2/54 (3.7%)	0.238	1.09 (0.22–5.40)
**GDM**†	287/4655 (6.2%)	7/54 (13.0%)	0.079	1.47 (0.61–3.56)
**Abruption**†	26/4655 (0.6%)	1/54 (1.9%)	0.268	1.53 (0.17–13.45)
**Fetal death in Utero (n = 4746)**	22/4692 (0.5%)	3/54 (5.6%)	<0.005	6.61 (1.63–26.82)
**SGA (n = 4665)**	295/4616 (6.4%)	3/49 (6.1%)	1.000	1.08 (0.32–3.61)

pPROM, preterm premature rupture of membrane; GDM, gestational diabetes mellitus; SGA, small for gestational age.

* adjusted for maternal age, BMI, parity and assisted reproduction.

† The obstetric outcomes were not available in 37 patients who were delivered in other institutions.

Table 4 describes the characteristics of women with vanishing twin delivered in preterm period. The causes of sPTB were pPROM (n = 4) and PTL (n = 1), and the causes of iPTB were FDIU (n = 3) and preeclampsia (n = 1). Specifically, all PTB in vanishing twin occurred in pregnant women who conceived after assisted reproduction.

**Table 4:** Characteristics of women with vanishing twin delivered in preterm period.

No.	Age	Parity	Mode of conception	Pre-BMI	GA of vanishing twin	Chorionicity	cGAD	Cause of Preterm birth
1	36	Nulli	ART	22.5	6.00	Unknown	16.29	FDIU
2	41	Nulli	ART	22.2	7.29	DCDA	22.71	FDIU
3	39	Nulli	ART	25.3	11.00	Unknown	25.43	FDIU
4	37	Multi	ART	19.0	7.00	DCDA	26.29	Preeclampsia
5	31	Nulli	ART	21.1	10.00	DCDA	17.43	pPROM
6	37	Nulli	ART	18.9	9.00	DCDA	27.86	pPROM
7	36	Nulli	ART	26.5	5.50	Unknown	32.86	pPROM
8	35	Nulli	ART	43.7	8.00	DCDA	15.57	pPROM
9	37	Nulli	ART	23.2	7.00	DCDA	35.00	PTL

BMI, body mass index; GA, gestational age; GAD,; ART, assisted reproductive technology; DCDA, dichorionic diamniotic; FDIU, fetal death in utero; pPROM, preterm premature rupture of membrane.

Therefore, we sub-analyzed the risk of preterm birth in vanishing twin according to the etiology of PTB (Table 5). The risk of PTB increased only in pregnant women with vanishing twin who conceived after assisted reproduction. The pregnant women with vanishing twin who spontaneously conceived were not at increased risk for preterm birth.

**Table 5:** Risk of preterm birth according to mode of conception.

	ART			Natural pregnancy		
	Singleton (n = 528)	Vanishing twin (n = 40)	P	Singleton (n = 4,164)	Vanishing twin (n = 14)	P
**Preterm birth**						
**<28 wks**	12 (2.3%)	7 (17.5%)	<0.001	31 (0.7%)	0 (0%)	1.000
Spontaneous PTB <28 wks	6 (1.1%)	3 (7.5%)	<0.05	11 (0.3%)	0 (0%)	1.000
Indicated PTB <28 wks	6 (1.1%)	4 (10.0%)	<0.005	20 (0.5%)	0 (0%)	1.000
**<32 wks**	16 (3.0%)	7 (17.5%)	<0.005	57 (1.4%)	0 (0%)	1.000
Spontaneous <32 wks	9 (1.7%)	3 (7.5%)	<0.05	24 (0.6%)	0 (0%)	1.000
Indicated PTB <32 wks	7 (1.3%)	4 (10.0%)	<0.05	33 (0.8%)	0 (0%)	1.000
**<34 wks**	22 (4.2%)	8 (20.0%)	<0.005	77 (1.8%)	0 (0%)	1.000
Spontaneous <34 wks	13 (2.5%)	4 (10.0%)	<0.05	36 (0.9%)	0 (0%)	1.000
Indicated PTB <34 wks	9 (1.7%)	4 (10.0%)	<0.05	41 (1.0%)	0 (0%)	1.000
**<37 wks**	58 (11%)	9 (22.5%)	0.40	268 (6.4%)	0 (0%)	1.000
Spontaneous <37 wks	38 (7.2%)	5 (12.5%)	0.214	185 (4.4%)	0 (0%)	1.000
Indicated PTB <37 wks	20 (3.8%)	4 (10.0%)	0.080	83 (2.0%)	0 (0%)	1.000

ART, assisted reproductive technology.

## Discussion

The principal findings of the current study are as follows: 1) Vanishing twin group had higher risk for total PTB (<34 weeks, <32 weeks, and <28 weeks) than singleton group; 2) In terms of etiology of PTB, the vanishing twin group had increased risk for both sPTB and iPTB (<34 weeks, <32 weeks, and <28 weeks), and this increased risk for sPTB and iPTB in VT group remained significant even after controlling for confounding variables; 3) Cases with vanishing twin had higher risk for fetal death in utero, and this difference remained significant after adjustment for confounding variables; and 4) An increased risk of PTB was noted in only pregnancies conceived after ART.

There have been several reports on the risk of preterm birth in vanishing twin, and the results are inconsistent (Table 6). A few studies reported an increased risk of preterm birth before 37 weeks [3, 6, 15], 34 weeks [5, 6], 32 weeks [3, 15] and 28 weeks [5] of gestation. However, La Sala and Phillip reported that the preterm birth was not increased in vanishing twin [7, 11]. Recently, a meta-analysis of the obstetric outcome of vanishing twin has been performed [16], and preterm birth less than 34 weeks was significantly increased in vanishing twin group. In the current study, we have also shown that the risk of preterm birth before 34, 32, and 28 weeks of gestation was increased in the vanishing twin group.

**Table 6:** Previous studies and the current study regarding the risk of preterm birth in vanishing twin.

	Almog et al. [5]	Chasen et al. [15]	La Sala et al. [7]	Pinborg et al. [3]	Shebl et al. [6]	Romanski et al. [11]	The current study
Years	1999–2007	2003–2005	1992–2004	1995–2001	1999–2005	2007–2015	2016–2018
ART status	IVF with or without ICSI	IVF	IVF with or without ICSI	IVF with or without ICSI	IVF with or without ICSI	IVF	ALL
The number of vanishing twin	57	55	84	642	46	100	54
The number of control singleton pregnancy	171	168	602	5237	92	798	4692
< 37weeks		12.7% vs 8.9% (p = 0.44)	16.7% vs 15.9% NS	13.2% vs 9.0% (p<0.001)	19.6% vs 8.7% (p = 0.067)	17% vs 14.8% NS	16.7% vs 6.9% (p = 0.076)
< 34weeks	22.8% vs 5.8% (p = 0.0003)				4.3% vs 2.2% (p = 0.47)		14.8 vs 2.1% (p<0.001)
< 32weeks		7.3% vs 1.8% (p = 0.06)	2.4% vs 2.5% (p = 1.0)*	3.8% vs 1.3% (p<0.001)			13% vs 1.6% (p<0.001)
< 28weeks	7.0% vs 1.2% (p = 0.01)						13% vs 0.9% (p<0.001)

IVF, in vitro fertilization; ICSI, Intracytoplasmic sperm injection.

*including 32weeks.

The current study has several strengths compared to previous studies. First, this study shows that the vanishing twin group is at increased risk not only for total preterm birth but also for sPTB and iPTB. To our knowledge, this is the first study that analyzed the risk of PTB according to the etiology of preterm birth. In this study, we divided the etiology of preterm birth into sPTB (preterm labor, preterm premature rupture of membranes, or cervical insufficiency) and iPTB (PTB because of maternal or fetal indication).

Second, the current study included all pregnancies, regardless of the mode of conceptions, in contrast to the previous studies that excluded natural pregnancy and analyzed the obstetric outcomes only in women who became pregnant through ART such as IVF only or IVF/ICSI [3, 5–7, 15, 17]. The result shows that the risk of PTB in vanishing twin was increased only in pregnant women who conceived after ART, not in naturally pregnant women. To our knowledge, this is also the first study that compared the risk of PTB in vanishing twin according to the mode of conception. However, the number of cases with natural pregnancy is relatively small to conclude this issue. More studies are needed to explain this discrepancy in results according to the mode of conception.

Lastly, compared to previous retrospective cohort studies, our study was a prospective cohort study, which enrolled pregnant women from early pregnancy up to the time of delivery; therefore, we could determine the specific cause of preterm birth.

Then, why both sPTB and iPTB are increased in vanishing twin? There were several previous studies that vanishing twin potentially affects a substantial risk for congenital malformations [18]. However, the reason for the higher frequency of preterm birth in vanishing twin is still uncertain. In the current study population, the main causes of preterm birth in vanishing twin were pPROM (80% in spontaneous PTB) and FDIU (75% in indicated PTB). Several possible mechanisms can be considered. First, both FDIU and pPROM might be because of subsequent inflammation in vanishing twin. This inflammatory condition may affect the intra-uterine environment, resulting in placental under-perfusion or placental inflammation. There are substantial evidences that inflammation is the main cause in the pathogenesis of not only pPROM but also fetal death [19–21]. To determine the relationship between inflammatory condition and the subsequent risk of PTB in vanishing twin, further studies should include some objective criteria such as inflammatory cytokines. Second, it is well known that coagulation cascades are triggered in the death of one fetus in twin pregnancy [22]. Even in vanishing twin, pro-coagulant condition may be evoked in the intra-uterine environment, resulting in impairment of uteroplacental perfusion. Third, in monochorionic twin, vascular anastomosis may affect the remained fetus [18]. Also, according to a previous study, monochorionic twins have significant risk in the presence of one fetal loss than dichorionic twins [23]. However, this pathogenesis is unlikely according to the current study, because pPROM or FDIU occurred not only in monochorionic twin but also in dichorionic twin.

There are several points to be considered. First, the original study design was to analyze the cost-effectiveness of various prenatal test strategies for Down syndrome, and the relationship between vanishing twin and the risk of PTB was not included as a part of the prospective study. Second, it is our routine practice to perform as early as possible to confirm intrauterine pregnancy. But the first ultrasound examination may not be performed at 6–8 weeks of gestation in all of the cases, and there is the possibility of allocation bias in the current study. Further prospective studies regarding this issue are needed to confirm the result of the current study.

## Conclusion

In conclusion, vanishing twin can be an independent risk factor for both sPTB and iPTB when compared with singleton pregnancy.

## Supporting information

S1 Data (SAV)
